# A novel multistage antigens ERA005f confer protection against *Mycobacterium tuberculosis* by driving Th-1 and Th-17 type T cell immune responses

**DOI:** 10.3389/fimmu.2023.1276887

**Published:** 2023-11-07

**Authors:** Xueting Fan, Xiuqin Zhao, Ruibai Wang, Machao Li, Xiuli Luan, Ruihuan Wang, Kanglin Wan, Haican Liu

**Affiliations:** ^1^ National Key Laboratory of Intelligent Tracking and Forecasting for Infectious Disease, National Institute for Communicable Disease Control and Prevention, Chinese Center for Disease Control and Prevention, Beijing, China; ^2^ Department of Tuberculosis, Beijing Chest Hospital, Capital Medical University/Beijing Tuberculosis and Thoracic Tumor Research Institute, Beijing, China

**Keywords:** *Mycobacterium tuberculosis*, multistage subunit vaccine, Th-1 immunity, Th-17 immunity, alum adjuvant

## Abstract

**Introduction:**

Tuberculosis (TB) is a major threat to human health. In 2021, TB was the second leading cause of death after COVID-19 among infectious diseases. The Bacillus Calmette–Guérin vaccine (BCG), the only licensed TB vaccine, is ineffective against adult TB. Therefore, there is an urgent need to develop new effective vaccines.

**Methods:**

In this study, we developed a novel multistage subunit vaccine (ERA005f) comprising various proteins expressed in metabolic states, based on three immunodominant antigens (ESAT-6, Rv2628, and Ag85B). We utilized the *E. coli* prokaryotic expression system to express ERA005f and subsequently purified the protein using nickel affinity chromatography and anion exchange. Immunogenicity and protective efficacy of ERA005f and ERA005m were evaluated in BALB/c mice.

**Results:**

ERA005f was consistently expressed as an inclusion body in a prokaryotic expression system, and a highly pure form of the protein was successfully obtained. Both ERA005f and ERA005m significantly improved IgG titers in the serum. In addition, mice immunized with ERA005f and ERA005m generated higher titers of antigen-specific IgG2a than the other groups. Elispot results showed that, compared with other groups, ERA005f increased the numbers of IFN-γ-secreting and IL-4-secreting T cells, especially the number of IFN-γ-secreting T cells. Meanwhile, ERA005f induced a higher number of IFN-γ^+^ T lymphocytes than ERA005m did. In addition, ERA005f improved the expression of cytokines, including IFN-γ, IL-12p70, TNF-α, IL-17, and GM-CSF and so on. Importantly, both ERA005f and ERA005m significantly inhibited the growth of Mtb.

**Conclusion:**

The novel multistage antigen ERA005f elicited a strong antigen-specific humoral response and Th-1 and Th-17 cell-mediated immunity in mice. Meanwhile, it can effectively inhibit H37Rv growth *in vitro*, and represents a correlate of protection *in vivo*, indicating that ERA005f may exhibit excellent protective efficacy against *Mycobacterium tuberculosis* H37Rv infection. Our study suggests that ERA005f has the potential to be a promising multistage tuberculosis vaccine candidate.

## 1 Introduction

Even in the time of SARS-CoV-2, tuberculosis (TB), caused by *Mycobacterium tuberculosis* (Mtb), is the leading global infectious killer (1). According to the World Health Organization’s Global Tuberculosis Report 2021, approximately 9.9 million people were diagnosed with TB, and 1.3 million people died of TB in 2021. Despite the widespread use of standard drug regimens, modern diagnostics, and a vaccine (Bacille Calmette–Guerin vaccine, BCG), the global tuberculosis epidemic is inadequately controlled. BCG provides effective protection against severe forms of TB in infants and children, although it shows variable efficacy against pulmonary TB in adults (2). To date, a number of new TB vaccines have been studied, including recombinant BCG (rBCG), adjuvanted protein subunit vaccines, viral vector-delivered subunit vaccines, DNA vaccines, and RNA vaccines (3). Among these TB vaccine strategies, one aim is to replace BCG and the other is to develop a vaccine that could enhance or prolong the protection provided by BCG (4, 5). Recently, several vaccines have been tested in clinical and preclinical trials. Unfortunately, although these vaccines showed greater improvement than BCG in mice, very few of them showed perfect protective immunity in trials (6, 7). For these reasons, an effective novel TB vaccine should be developed to reduce or eradicate TB, which will play a pivotal role in achieving the goal of ending the TB epidemic by 2035.

Subunit vaccines have emerged as a prominent research area for novel tuberculosis vaccines owing to their inherent advantages of safety, stringent standards, cost-effectiveness, and ease of transportation (8–10). In recent years, substantial progress has been made in TB subunit vaccines, with several vaccines in clinical trials, such as MVA85A, M72/AS01, and H51:IC31 (1). Previous studies have demonstrated that filtrate antigens provide protection similar to BCG (8, 11, 12). However, the protective efficacy of single-antigen subunit vaccines in animal models has been limited, as only a few have demonstrated success thus far. Moreover, the M72/AS01 vaccine exhibited 54% protective efficacy, attributed to the presence of two highly immunogenic antigens from Mycobacterium tuberculosis, namely Mtb32A and Mtb39A. This finding highlights its potential as an innovative subunit vaccine for tuberculosis. Recent research on subunit vaccines suggests that an ideal TB vaccine should consist of proteins or multiple epitopes at various stages of infection.

Mtb is an intracellular bacterium that has been able to host macrophages for several years. During Mtb infection, the infective bacteria include varying growing, slow growing, and non-growing subpopulations, and the subpopulations, which can interconvert with each other (13). TB vaccines not only provide protection against replicating bacteria, but also attack dormant bacteria (14, 15). Thus, an ideal subunit TB vaccine should consist of multi-stage Mtb antigens.

In this study, we developed a novel multistage vaccine consisting of three antigens, ESAT-6 (Rv3875), Rv2628, and Ag85B (Rv1886c). ESAT-6, a virulence factor of Mtb, is an early secreted antigen that acts independently or in combination with CFP10 to modulate host immune responses (16, 17). Previous studies have shown that vaccines containing ESAT-6 can provide protection efficacy against TB (15, 18, 19). Recent studies have shown that ESAT-6 induces strong T cell-mediated immunity (6). Ag85B is an early secreted antigen associated with active bacterial replication (20, 21). It consists of the Ag85 complex, capable of eliciting T-cell responses in both humans and mice, and particularly increases the levels of Th1 cytokines (such as IFN-γ) (6). Rv2628 is an Mtb latency-associated antigen, eliciting a strong humoral immune response and a higher proportion of CD4+CD25+ or CD4+Foxp3+ T cells (22). These studies demonstrated that these antigens were highly immunogenic and provided protective immunity, suggesting that they are promising candidates for TB vaccines. In this study, we developed a new multistage vaccine called ERA005f, which consists of ESAT-6-Rv2628-Ag85B linked by a GGSGG linker with an affinity tag. The fusion protein was combined with an alum adjuvant and its immunogenicity and protective effects were evaluated in BALB/c mice.

## 2 Materials and methods

### 2.1 Ethics statement

All animal experiments were performed in accordance with the Animal Experimental Ethical Committee of the National Institute for Communicable Disease Control and Prevention, Chinese Center for Disease Control and Prevention (permit number. 2021-0225).

### 2.2 Construction, expression, and purification of the fusion protein

**Figure 1:**
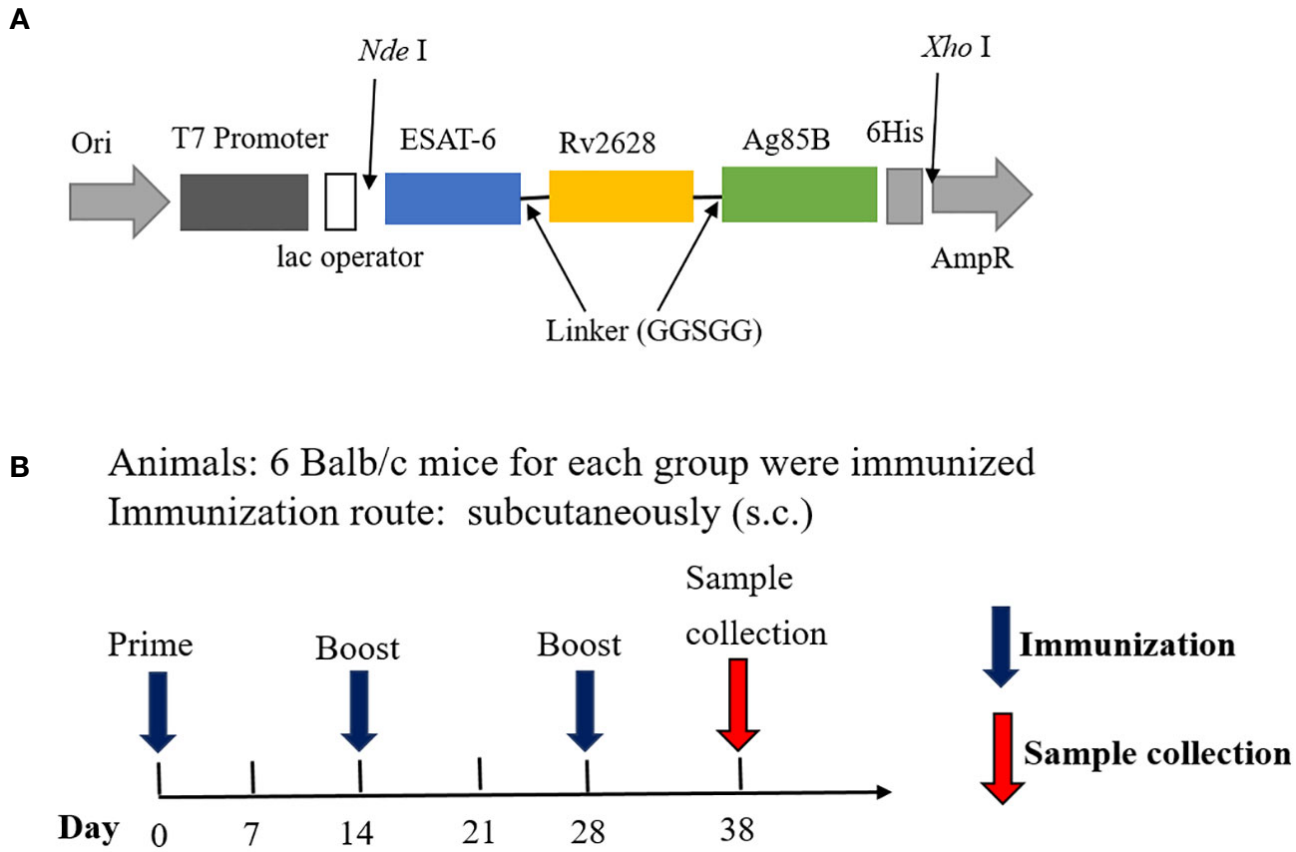
Structural diagram of the fusion protein ERA005f and vaccination schedule. **(A)** Structure of ERA005f. **(B)** The schedule of vaccination and sample collection.

The recombinant prokaryotic expression plasmid was constructed by inserting the synthesized gene sequence ESAT-6-Rv2628-Ag85B, which is a flexible-linked fusion of different antigens with a C-terminal 6His-tag, into pET43.1a (**Figure 1A**), this fusion protein was named ERA005f. Additionally, we constructed three single-antigen recombinant prokaryotic expression plasmids with a His-tag, namely pET32a-ESAT-6, pET32a-Rv2628, and pET32a-Ag85B. The recombinant plasmids were transferred into *Escherichia coli* BL21(DE3), and the expression of fusion proteins was induced with isopropyl β-D-thiogalactopyranoside (IPTG). After induction, the cells were harvested by centrifugation and suspended in lysis buffer (10 mmol/L Tris–HCl (pH = 8.0), 0.5% Triton-X100). The suspended samples were sonicated and SDS-PAGE was performed to analyze the expression of recombinant proteins. The four recombinant proteins were purified using the same procedure. First, the recombinant proteins were purified by nickel column chromatography as previously described (23). Subsequently, DEAE ion-exchange chromatography was performed to purify the proteins and obtain a higher level of purification. Endotoxin levels were detected using a Pierce Chromogenic Endotoxin Quant Kit (Thermo Fisher).The concentration of purified proteins was determined using a bicinchoninic acid (BCA) protein assay kit. The proteins were stored at −70°C until further use.

### 2.3 Vaccine preparation and immunization procedure

In this study, an alum adjuvant was used to assess the immunogenicity of antigens. Specific pathogen-free female BALB/c mice (8 weeks old) were obtained from Beijing HFK Bioscience Co. Ltd., (Beijing, China) and maintained in a Biosafety Level 2 laboratory while being fed standard laboratory chow. Twenty-four mice were divided into four groups: ERA005f/alum adjuvant, ESAT-6/Rv2628/Ag85B (ERA005m)/alum adjuvant, alum adjuvant, and PBS (n = 6) (**Table 1**; **Figure 1B**). Control mice were subcutaneously (s.c.) vaccinated with either PBS or alum adjuvant. ERA005f/alum adjuvant group comprised of 50 μg recombinant ERA005f protein and 1/3 volume of alum adjuvant. The ERA005m/alum adjuvant group consist of 50 μg mix antigens plus 1/3 volume of alum adjuvant, the molar mass proportion of ESAT-6, Rv2628, and Ag85B is 1:1:1. These groups were thenimmunized subcutaneously. All mice received three immunizations, with a 2-week interval between each one, and were sacrificed 10 days after the final immunization to collect samples for detection

**Table 1:** Immunizations.

Group	Adjuvant	Antigen	Route
A	–	PBS	subcutaneously
B	Alum	–	subcutaneously
C	Alum	ERA005f	subcutaneously
D	Alum	ERA005m	subcutaneously

### 2.4 The detection of antigen-specific IgG and IgG subclass antibodies

Antigen-specific IgG, IgG1, and IgG2a antibodies were determined using ELISA, as previously described (23). Briefly, 96-well plates were coated with 200 ng/well of ERA005f or ERA005m and incubated overnight at 4°C. The plates were then blocked with 5% (w/v) defatted milk in washing buffer at 37°C for 2 h. Serum samples were diluted two-fold and added to ELISA plates for incubation at 37°C for 2 h. HRP-conjugated goat anti-mouse IgG (1:2,000), IgG1(1:500), and IgG2a (1:500) were added, followed by an additional one-hour incubation period at 37°C. TMB substrate was added and incubated at room temperature for 20 min. The reaction was stopped with 2M H_2_SO_4_, and the optical density was measured at 450 nm.

### 2.5 Multiplex cytokines assay

Splenic lymphocytes were harvested using red blood cell lysis buffer (Solarbio, Beijing, China) and mouse lymphocyte separation medium (Dakewe, Beijing, China). After determining cell viability, cells were seeded at 2 × 10^5^cells/well in a 96-well plate. Subsequently, the cells were stimulated with ERA005f or ERA005m (5 μg), and supernatants were collected at 18 h to quantify cytokine levels. The levels of murine TNF-α, GM-CSF, IFN-γ, IL-12, IL-2, IL-10, IL-6, IL-4, and IL-17 were quantified using the Luminex technology and reagents (R&D Systems, Minneapolis, MN, USA).

### 2.6 ELISPOT assay

*Mycobacterium tuberculosis* antigen-specific T cell immune responses were assessed in mice using the ELISPOT assay. The mice were euthanized on day 10 after the final immunization. Individual splenocytes were prepared as described in *Section 2.5*. IFN-γ-secreting and IL-4-secreting T cells were detected using a mouse pre-coated ELISPOT kit (Dakewe, China) according to the manufacturer’s instructions.

### 2.7 *In vitro Mycobacterium* growth inhibition assays

To assess the efficacy of the vaccine, an unbiased approach was employed using mycobacterium growth inhibition assays (MGIAs) as previously described (24–26). Splenic lymphocytes were obtained as described in *Section 2.5*. Cells were seeded in six-well plates at a density of 1 × 10^6^ cells per well and Mtb H37Rv were collected and washed twice in 1× phosphate-buffered saline (PBS) containing 0.05% Tween-80, pelleted, and thoroughly resuspended in RPMI1640 medium with 0.05% Tween-80. Cells were infected with H37Rv at a multiplicity of infection (MOI) of 5 for 1 h at 37°C to facilitate bacterial entry into the cells. Subsequently, the culture media were discarded, and the cells were washed thrice with 1× PBS before being incubated again in fresh medium. The cocultures were incubated in a BSL-3 laboratory at 37°C for 96h with 5% CO_2_. After the incubation period, the co-cultures were transferred to 1.5 ml tubes and centrifuged at 12,000 rpm for 10 min, followed by careful removal of the supernatant. To measure H37Rv survival in lymphocytes, the cells were lysed in 7H9 broth containing 0.05% SDS for 10 min. Bacterial colony-forming units (CFUs) were determined by serial dilution plating on 7H10 agar plates, and bacterial numbers were counted using CFU.

### 2.8 Statistical analysis

Statistical significance for comparisons of multiplex groups was determined using one-way ANOVA (GraphPad Prism 9.0). In all experiments, statistical significance was set at P<0.05.

## 3 Results

### 3.1 Expression and purification of recombinant proteins

**Figure 2:**
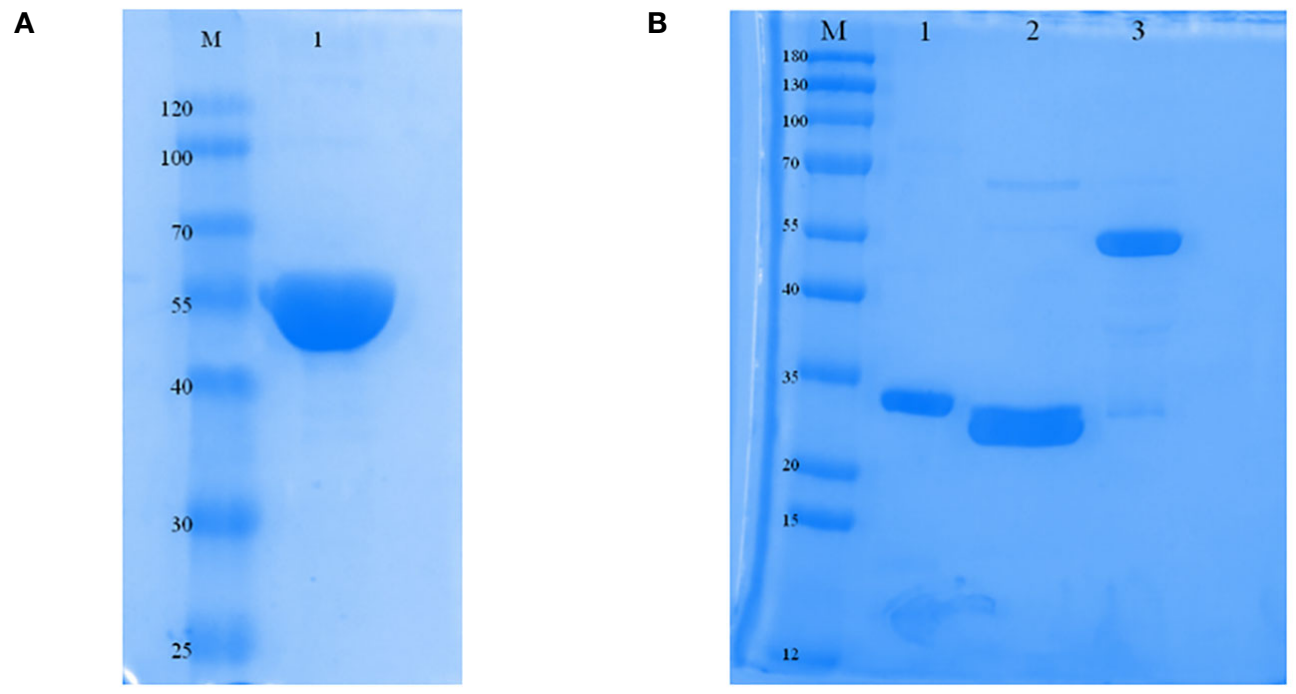
Expression and characterization of ERA005f and the multistage antigens. **(A)** Identification of purified ERA005f. **(B)** Identification of each purified multistage antigen. Lane1, purified Rv3875; Lane 2, purified Rv2628; Lane 3, purified Rv1886c.

The protein was primarily found in inclusion bodies, and the molecular mass of the ERA005f protein was found to be approximately 55 kDa by SDS-PAGE. The purified protein was successfully refolded and showed only one band with the expected molecular mass and a purity greater than 90% (**Figure 2A**). In addition, proteins such as ESAT-6, Rv2628, and Ag85B have all been purified for immunization (**Figure 2B**), and their molecular weights were 30.85 kDa, 33.71 kDa, and 56.26 kDa, respectively. In addition, endotoxin levels of the four proteins were lower than 0.5 EU/ml.

### 3.2 Similar patterns of antibody response elicited by subunit vaccines adjuvanted with alum

**Figure 3:**
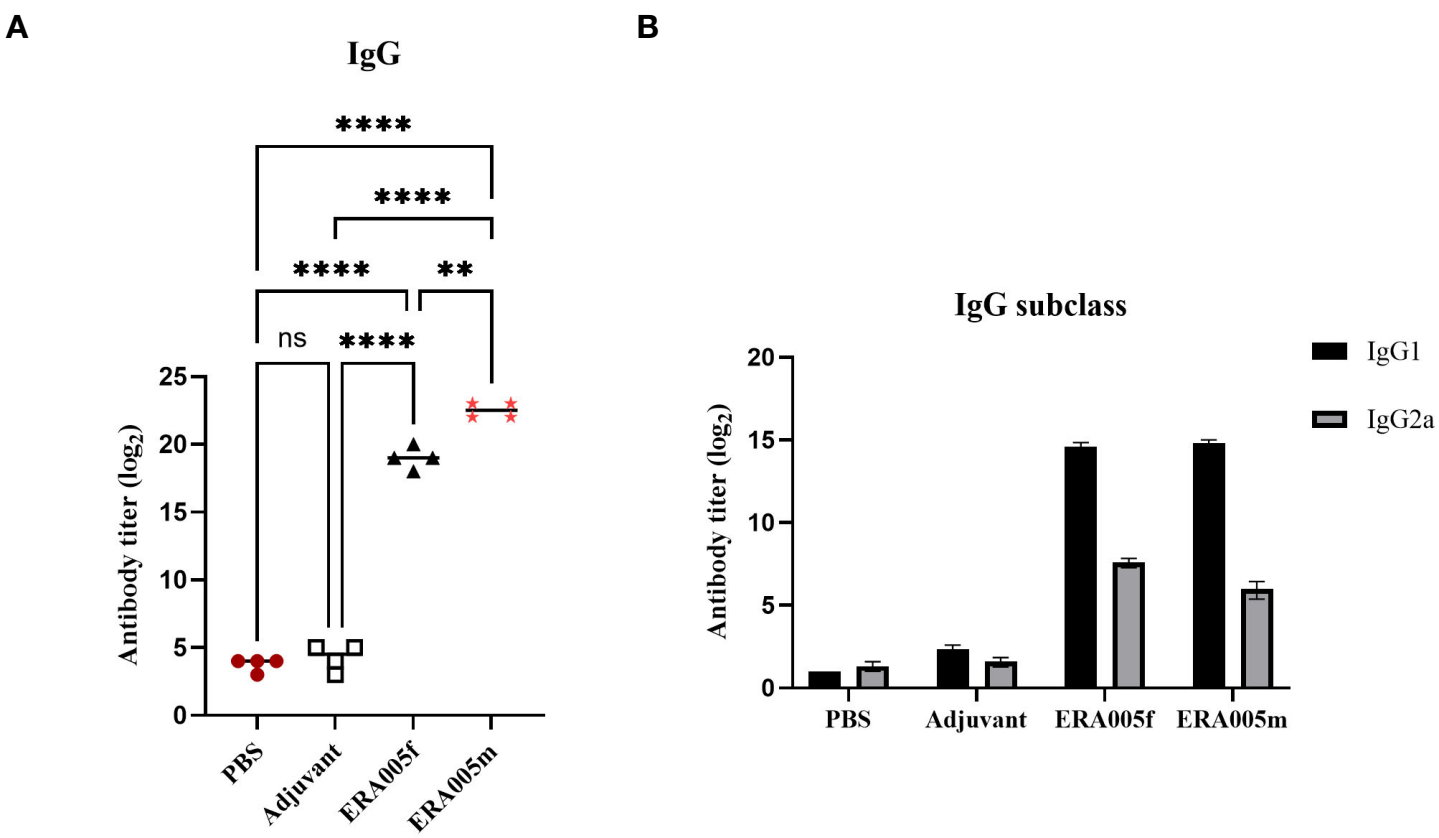
Levels of serum IgG, IgG1, and IgG2a antibodies against ERA005f in the different groups. Groups of BALB/c mice were immunized subcutaneously with PBS, alum as the adjuvant, or alum adjuvanted withERA005f or ERA005m. Ten days after the last immunization, antigen-specific antibody titers were detected using ELISA. **(A)** Antigen-specific IgG antibody titers in the serum. **(B)** Serum antigen-specific IgG1 and IgG2a antibodies. Results are presented as the mean ± SD, n = 4. ns means no significance, *P*>0.05, **P<0.01, ****P<0.0001, (unpaired student-t-test).

As expected, ERA005f and ERA005m significantly increased IgG titers in the serum after the final immunization compared to PBS and adjuvant controls (*P<*0.005). Importantly, alum-adjuvanted ERA005m immunizations led to a greater elevation of antigen-specific IgG in serum and was significantly greater than that with ERA005f plus alum (p<0.05) (**Figure 3A**).

### 3.3 ERA005f promoted the differential cytokine profiles

**Figure 4:**
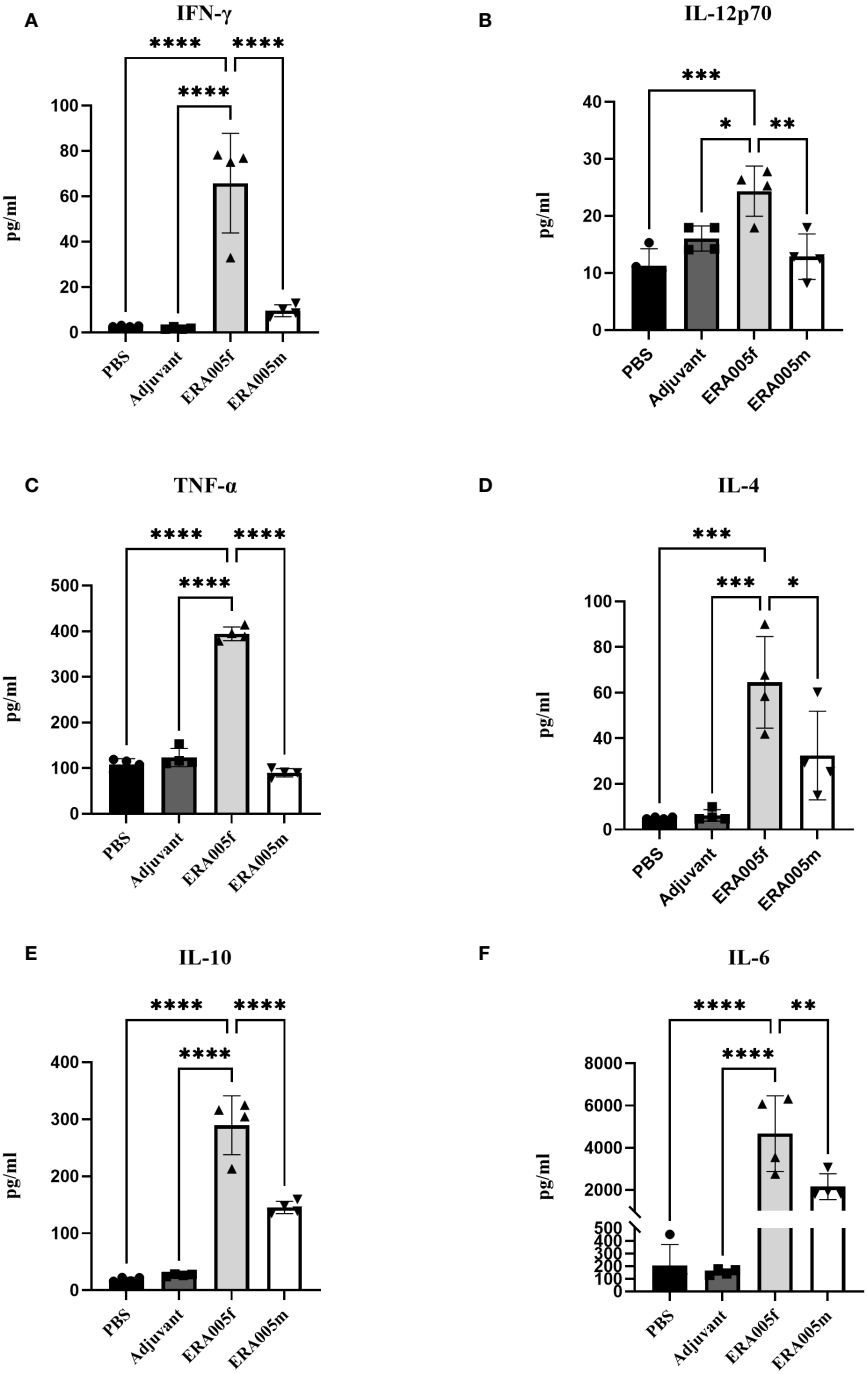
Induction of Th1 and Th2 type cytokines by different vaccines. Groups of BALB/c mice were immunized subcutaneously with PBS, alum as the adjuvant, or alum adjuvanted withERA005f or ERA005m. Th1 and Th2 type cytokines were measured in splenocyte samples collected 10 days after the last immunization. **(A–F)** Concentrations of IFN-γ, IL-12p70, TNF-α, IL-4, IL-10, and IL-6 (measured in pg/ml). Bars represent mean ± SD, n = 4. Asterisks indicate statistical significance between two groups, where *P<0.05, **P<0.01, ***P<0.001, and ****P<0.0001 using one-way ANOVA with Tukey’s multiple comparison tests.

**Figure 5:**
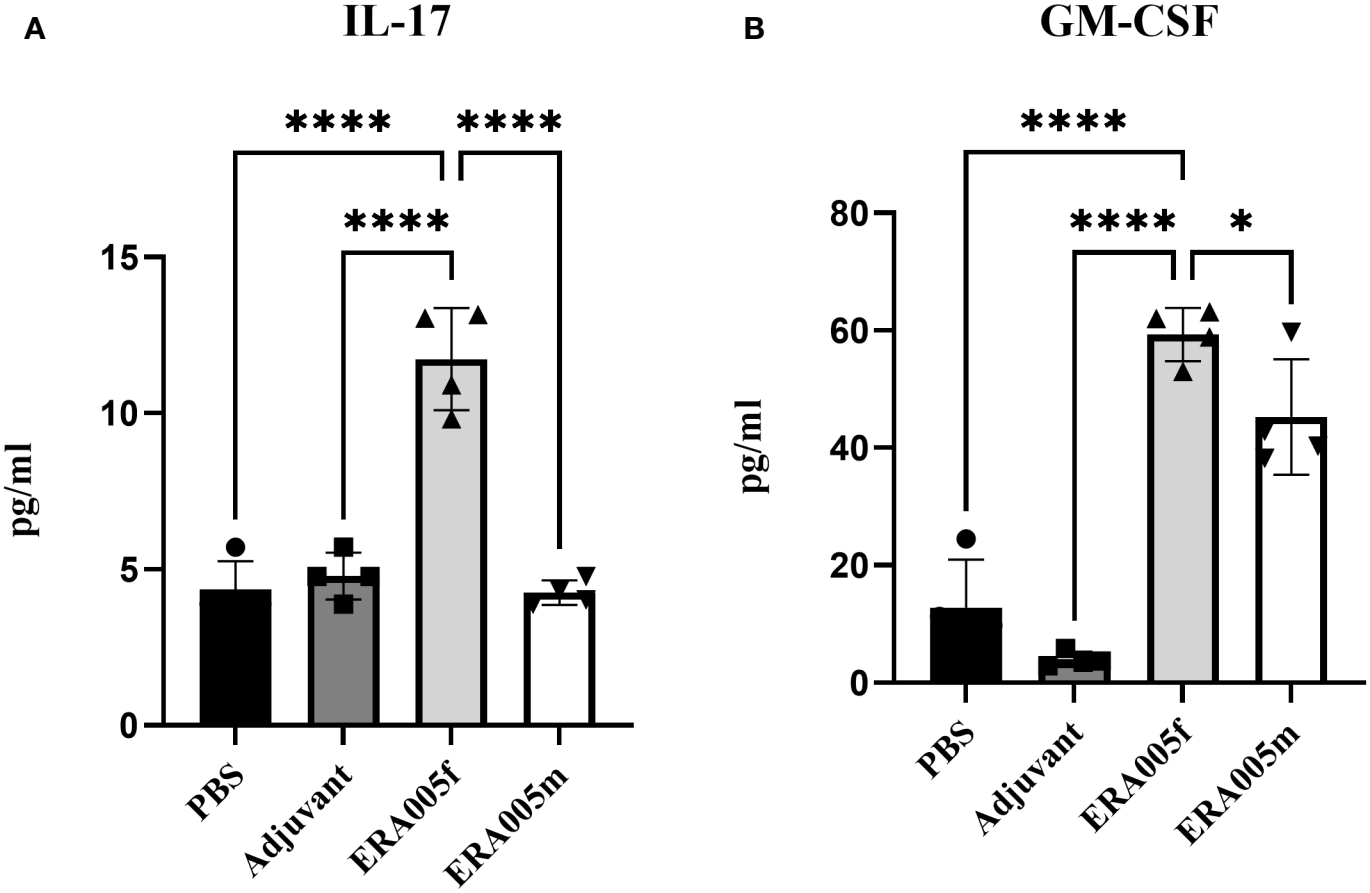
ERA005f drives robust Th17 type immune response. The mice were immunized subcutaneously with PBS, alum adjuvant alone, alum plus ERA005f, or ERA005m. The mice were immunized subcutaneously three times at 0-, 2-, and 4-week intervals. Ten days after the last booster vaccination, the spleen cells were stimulated with antigens ex *vitro*. IL-17 **(A)** and GM-CSF **(B)** were measured using Luminex. Values are mean ± SD (n = 4), *P<0.05, ****P<0.0001 by one-way ANOVA with Tukey’s multiple comparison test.

To investigate the effects of vaccine-induced T cell responses, we examined Th1/Th2/Th17 cytokine levels using Luminex assays. Data showed that both ERA005f and ERA005m enhanced cellular responses, particularly Th1 and Th17 type cytokines (**Figures 4**, **5**). Compared to PBS and adjuvant alone, the secretion of IFN-γ was significantly increased in the groups vaccinated with antigen plus alum. The production of IFN-γ in mice vaccinated with ERA005f plus alum was approximately 6-fold higher than that in mice vaccinated with ERA005m plus alum (**Figure 4A**, p<0.01). Furthermore, a significant elevation in other Th-1 cytokines, IL12p70 (**Figure 4B**) and TNF-α (**Figure 4C**), was observed following immunization with ERA005f and ERA005m, as compared to the PBS group. Significantly, ERA005f immunization induced a noteworthy increase in the secretion of IL-12p70 and TNF-α compared to the ERA005m group, with a statistically significant difference observed (p<0.05 and p<0.001, respectively). These cytokines play a prominent role in the prevention of tubercolosis. Nonetheless, there was no statistically significant change in the levels of IL-2 (data not shown). In addition, ERA005f significantly increased the levels of Th17 cytokines IL-17 and GM-CSF (**Figure 5**, p<0.05), with the level of IL-17 being notably higher than that observed in the ERA005m group (p<0.001). In addition, the levels of Th2 cytokines IL-10 (**Figure 4E**) and IL-6 (**Figure 4F**) were also significantly elevated in ERA005f immunized mice as compared to those in the ERA005m group.

### 3.4 ERA005f induces Th1 biased immune response

**Figure 6:**
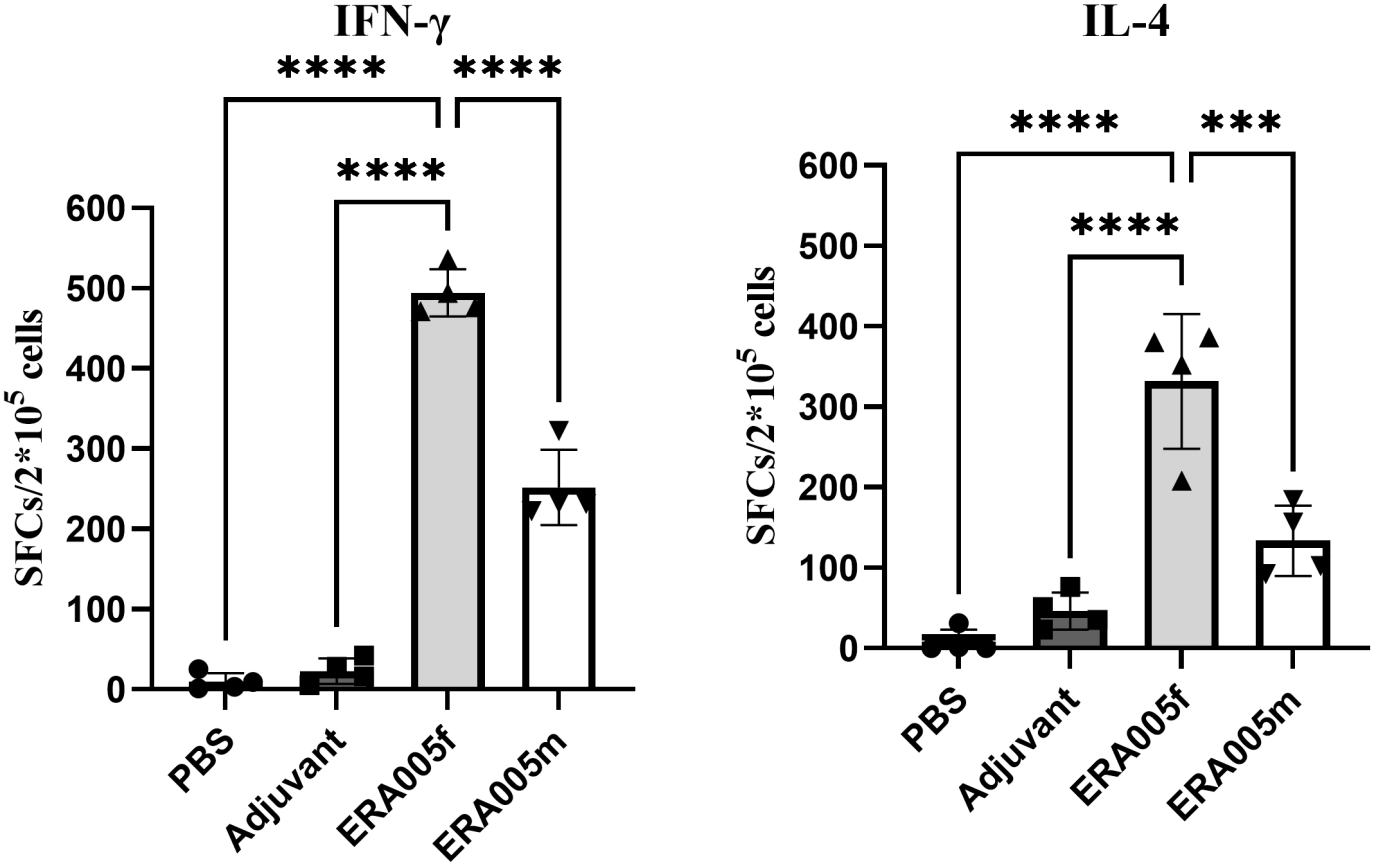
Antigen-specific IFN-γ and IL-4 secretion in the splenocytes of mice immunized with different vaccines. Mice were immunized subcutaneously with PBS, alum adjuvant alone, alum plus ERA005f or ERA005m. The mice were immunized subcutaneously three times at 0-, 2-, and 4-week intervals. Ten days after the last booster vaccination, spleen cells were stimulated with antigens *in vitro*. IFN-γ and IL-4 levels were detected using ELISPOT. The number of cells secreting IFN-γ/IL-4 is showed. The results are shown as the mean ± SD from groups of four mice. ***P<0.001, and ****P<0.0001.

To determine the IFN-γ/IL-4 secreting cells induced by the ERA005f and ERA005m vaccines, ELISPOT assays were performed on splenocytes from mice (**Figure 6**). The number of IFN-γ^+^ T lymphocytes (shown as SFCs in **Figure 6**) in mice vaccinated with ERA005f and ERA005m was significantly higher than that in the PBS group (**Figure 6A**). Similar results were observed for IL-4^+^ T lymphocytes (**Figure 6B**). However, the number of IFN-γ^+^ T lymphocytes was significantly higher after immunization with ERA005f than that with ERA005m. Notably, the frequency of IFN-γ-secreting T cells was significantly higher than that of IL-4 in the ERA005f immunized mice. These results unambiguously demonstrated that the combination of ERA005f and alum induces a Th1-biased immune response. Additionally, ERA005m induced a higher frequency of IFN-γ-secreting T cells than IL-4-secreting T cells, although the ratio was lower than that of ERA005f. This revealed that ERA005f enhanced the generation of robust Th1-type cellular immune responses.

The levels of antigen-specific IgG subtypes IgG1 and IgG2a were measured to determine the type of T cell response stimulated after immunization (**Figure 3B**). IgG 2a and IgG1 levels were measured to assess the polarization of the T-helper cell population. Previous studies have indicated that IgG subclasses reflect the T helper cell subset involved in immune response. In mice, IgG2a antibody levels may reflect a predominant Th1 type immune response, whereas the Th2 type immune response is related to the preferential induction of IgG1 (27). Mice immunized with ERA005f and ERA005m generated higher titers of antigen-specific IgG2a than the other groups (**Figure 3B**), indicating a T helper type 1 (Th1) response. Moreover, the IgG2a titer induced by ERA005f was significantly higher than that inERA005m.

### 3.5 ERA005f and ERA005m immunization induces mycobacterial growth inhibition in mouse splenocytes

**Figure 7:**
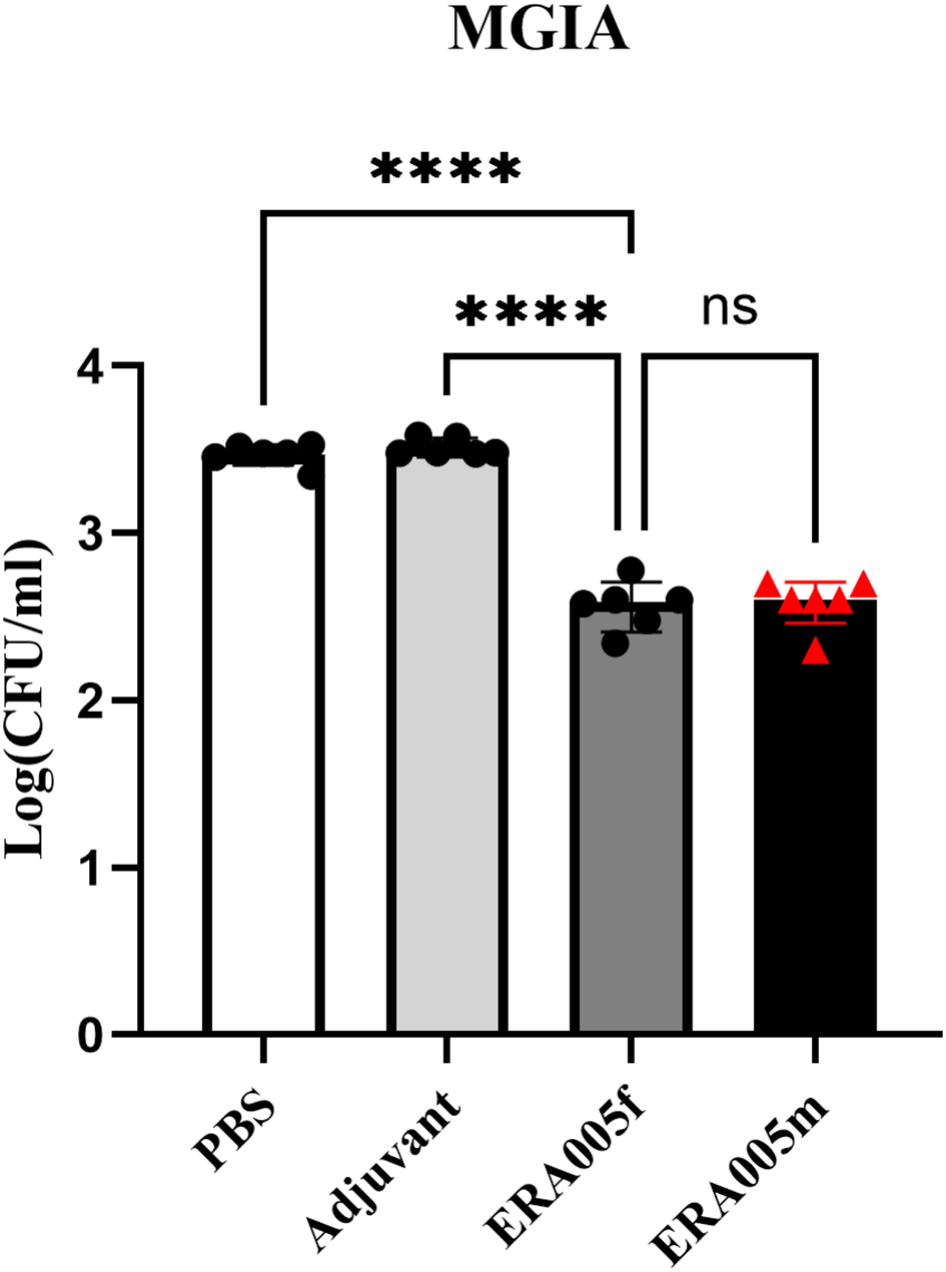
ERA005f immunization induced mycobacterial growth inhibition in murine splenocytes. Groups comprising six mice were euthanized 10 days after the final vaccination for utilization in the MGIA. Cells (1–2 × 10^6^) were infected with H37Rv at a multiplicity of infection (MOI) of 5 for 1 h at 37°C. Subsequently, the culture media were discarded, and the cells were washed thrice with 1× PBS before being incubated again in fresh medium for 96 h. Then cells were then lysed in 7H9 broth containing 0.05% SDS for 10 min. Bacterial colony-forming units (CFUs) were determined by serial dilution plating on 7H10 agar. The data presented represent the log10 CFU values for mice that were not immunized and those that received vaccines. Results were shown as mean±SD from groups of 6 mice. ns means no significance, *P*>0.05, ****P<0.0001.

The mycobacterial growth inhibition assay (MGIA) has been developed as a comprehensive tool to evaluate vaccine potency *ex vivo*, and MGIA results correspond to protection from Mtb challenge (28). The protective efficacy of ERA005f and ERA005m as subunit vaccines was evaluated using MGIAs. Splenocytes obtained from ERA005f and ERA005m immunized mice were better able to inhibit the growth of H37Rv in culture than splenocytes obtained from unimmunized animals (PBS or alum alone), with significantly reduced CFU (**Figure 7**, p<0.0001). Surprisingly, there were no statistically significant differences between the two groups. This revealed that ERA005f and ERA005m plus alum adjuvants could show protective functions and act as promising candidates for TB vaccines.

## 4 Discussion

Mtb is a multi-faceted pathogen that lacks host immunity according to numerous strategies, including rapidly growing and non-replicating dormant subpopulations that can interconvert with each other (13). The expressed antigens varied at different stages. Therefore, an optimal TB subunit vaccine should encompass diverse antigens from different bacterial stages to confer comprehensive protection against various Mtb stages.

To date, numerous novel TB vaccines comprising early and late expression antigens of Mtb have demonstrated immunogenicity and protective efficacy in animal models, such as LT70, LT69, and H1-IC31 (29, 30). In the present study, we constructed a novel fusion protein, ERA005f, based on three antigens (ESAT-6, Rv2628, and Ag85B), which are expressed at different stages of bacterial growth. ESAT-6 and Ag85B, which are early secreted antigens, have been identified as TB vaccine antigens. To the best of our knowledge, latent infections may constitute a significant reservoir for new cases of tuberculosis. In other words, latency antigens may constitute a pivotal element of the TB vaccine candidate antigens. Approximately 48 genes are involved in the expression of Mtb to form a dormancy survival regulator, or the DosR regulon (29). Rv2628 is a latency-associated antigen, which belongs to the group of eight DosR-regulated genes that are induced in response to stress, and cutokine-producing T cells specific for this antigen are observed after prolonged infection (30). We evaluated the immune response of multi-antigen fusion protein ERA005f in mice and found that mycobacterial growth in the MGIA was inhibited in the multistage vaccine ERA005f-vaccinated mice, suggesting that this novel antigen can produce an effective protective effect against *Mycobacterium tuberculosis* challenge (**Figure 7**).

The results showed that the fusion protein ERA005f was mainly expressed in the inclusion bodies. All the proteins in this study were successfully obtained by chromatography (**Figure 2**). To obtain effective vaccines for ERA005f and ERA005m, we examined the immunity and protection of these proteins, which are licensed in humans. In the present study, we observed the body weight and physiological status of the mice within two weeks of the prime immunization. As expected, the mice showed no weight loss or other clinical symptoms; therefore, the results are not shown.

Numerous studies have highlighted that cell-mediated immunity (CMI) is the main immune response against intracellular pathogens such as Mtb (31–33), especially the Th1-type immune response. Meanwhile, IFN-γ produced by T cells regulates immunity against TB (18). Specifically, Elispot results showed a significantly higher frequency of IFN-γ-producing antigen-specific CD4+T cells in the ERA005f group than in the other groups (**Figure 6**). Studies of recombinant BCG, DNA vaccine, or other subunit TB vaccines have confirmed that the expression of cytokines such as IFN-γ, TNF-α, and IL-2 can significantly improve protective efficacy (34–37). Meanwhile, mice receiving the ERA005f subunit vaccine generated high levels of antigen-specific IFN-γ as well as high levels of IgG1 and IgG2a antibodies. These mice exhibited a higher ratio of IgG2a/IgG1 than the other groups, indicating that ERA005f induced a Th1-type immune response. Secondly, Th17-related cytokines, including IL-17 and GM-CSF, as well as other cytokines such as IL-6, have been shown to promote the recruitment of Th1 cells and act as effector molecules against Mtb infection (38, 39). IL-12 is also critical for the induction of Th-1 mediated immunity against Mtb, promoting the growth and survival of Th-1 cells. In addition, some researchers have suggested that an ideal multi-stage subunit vaccine strongly induces Th1-type cytokines such as IFN-γ and TNF-α (9, 10).Vaccination with ERA005f resulted in remarkably increased expression of key cytokines, including IFN-γ, GM-CSF, IL-12p70, IL-6, TNF-α, and IL-17, which have been shown to contribute to Mtb control (40–42). These results indicated that the ERA005f mix with alum adjuvant not only promoted Th-1 type responses but also stimulated Th-17 type responses. However, these data merely suggest the potential of this antigen as a TB vaccine candidate and do not demonstrate its efficacy in protecting against mycobacterium tuberculosis infection.

In this study, we performed Mycobacterial Growth Inhibition Assays (MGIA) to provide evidence for the protection, which provides an unbiased measure of mycobacterial growth *in vitro* and may represent a functional correlation of protection (24, 26). Importantly, the multicomponent subunit vaccines ERA005f and ERA005m imparted significant protection compared to the other groups, reducing about 0.88Log_10_CFU than the PBS control (**Figure 7**, p<0.0001). Interestingly, the bacteriostatic effects of ERA005f and ERA005m showed a similar trend with no significant difference. We inferred that different stage antigens could provide better protection than a single antigen, whether these antigens were in a mixture or fusion form. Although antigen mixtures inhibited the survival of *Mycobacterium* H37Rv, they showed weaker Th1 and Th-17 immune response. Thus, our results suggest that the fusion expression of multiple antigens has a better immune effect than the antigen mixture. Therefore, we hypothesized that new epitope regions could be formed when multiple antigens are expressed in fusion, but not in mixtures alone, which could promote stronger cellular immunity.

In the present study, ERA005f elicited robust T cell and B cell responses and induced more Th-1 and Th-17 type cytokines. Although it can significantly inhibit the growth of Mtb H37Rv, this result was only in a mouse model, which is only an initial success. Our study also has some limitations, such as its immune effect in other animal models and the duration of immune protection are unknown, and whether the immune protective effect is better than that of BCG. Future work should compare the immune effects of ERA005f and BCG, and address how to optimally combine ERA005f with other immunization regimens to further optimize long-term protection against pulmonary tuberculosis in human adults and/or infants.

In summary, fusion protein ERA005f in alum adjuvant improved Th-1 and Th-17 type responses, especially the expression of IFN-γ, TNF-α, IL-17, and GM-CSF, and enhanced the protective efficacy inhibited Mtb growth in mice. Moreover, it elicits high levels of antigen-specific antibodies. These data suggest that ERA005f, which contains ESAT-6 (Rv3875), Rv2628, and Ag85B (Rv1886c), is a promising TB vaccine. In addition, our findings also confirmed that multiple antigens from different stages of Mtb infection may be a promising insight into TB vaccine design.

## Data availability statement

The original contributions presented in the study are included in the article/supplementary materials. Further inquiries can be directed to the corresponding authors.

## Ethics statement

The animal study was approved by The Animal Experimental Ethical Committee of the National Institute for Communicable Disease Control and Prevention, Chinese Center for Disease Control and Prevention. The study was conducted in accordance with the local legislation and institutional requirements.

## Author contributions

XF: Conceptualization, Data curation, Formal Analysis, Methodology, Project administration, Software, Validation, Writing – original draft, Writing – review & editing. XZ: Project administration, Writing – original draft. RBW: Project administration, Writing – original draft. ML: Validation, Writing – original draft. XL: Project administration, Writing – original draft. RHW: Software, Writing – original draft. KW: Conceptualization, Funding acquisition, Supervision, Writing – review & editing. HL: Conceptualization, Supervision, Writing – review & editing.
